# miR-155: An Important Role in Inflammation Response

**DOI:** 10.1155/2022/7437281

**Published:** 2022-04-06

**Authors:** Jingyan Hu, Songli Huang, Xiaoli Liu, Yuan Zhang, Shengli Wei, Xiuhua Hu

**Affiliations:** ^1^School of Life Sciences, Beijing University of Chinese Medicine, Beijing 102400, China; ^2^Xi'an Children's Hospital, Xi'an 710003, China; ^3^School of Chinese Materia Medica, Beijing University of Chinese Medicine, Beijing 102400, China

## Abstract

MicroRNAs (miRNAs) are a class of small, mature, noncoding RNA that lead to posttranscriptional gene silencing to regulate gene expression. miRNAs are instrumental in biological processes such as cell development, cell differentiation, cell proliferation, and cell apoptosis. The miRNA-mediated gene silencing is an important part of the regulation of gene expression in many kinds of diseases. miR-155, one of the best-characterized miRNAs, has been found to be closely related to physiological and pathological processes. What is more, miR-155 can be used as a potential therapeutic target for inflammatory diseases. We analyze the articles about miR-155 for nearly five years, review the advanced study on the function of miR-155 in different inflammatory cells like T cells, B cells, DCs, and macrophages, and then summarize the biological functions of miR-155 in different inflammatory cells. The widespread involvement of miR-155 in human diseases has led to a novel therapeutic approach between Chinese and Western medicine.

## 1. Introduction

**Figure 1:**
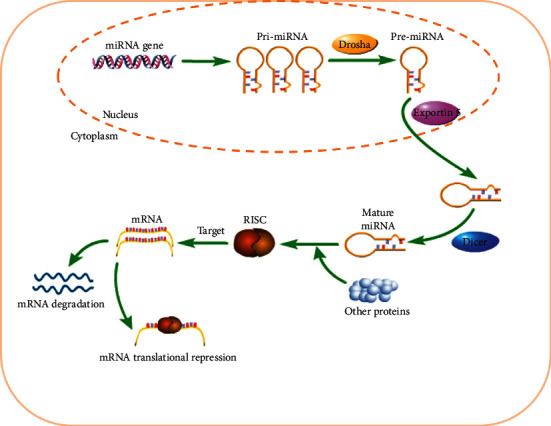
The forming process of microRNA.

MicroRNAs (miRNAs), initially identified in 1993, contained a family of small, noncoding RNAs that could be found in both introns and exons [1]. They act as adaptors for the miRNA-induced silencing complex to regulate posttranscriptional gene expression, activating Toll-like receptors and modulating protein production [2]. In mammalian cells, miRNAs usually guide the effector miRNA-induced silencing complex (miRISC) to bind with the 3′UTR of mRNAs, sometimes referred to as the miRNA-containing ribonucleoprotein particle (miRNP). Finally, miRNAs translation is inhibited, or miRNAs are degraded [3, 4]. The forming process of miRNAs is shown in Figure 1. With the advanced understanding of biology and the maturation in quantification methods, miRNAs are emerging as a biomarker of a specific pathology, such as vascular diseases, ophthalmic diseases, ocular diseases, cancer, and inflammation [5–8]. Therefore, making circulating miRNAs as biomarkers for inflammatory disease diagnosis and management has been researched for years [9]. Since circulating miRNAs may be active messengers, they may even trigger a systemic response to cell viability and even cell death, and they have the potential to be clinically relevant biomarkers for a number of physiopathological processes like inflammatory responses and inflammation-related conditions [10, 11].

## 2. MicroRNA-155(MiR-155)

miR-155 transcribed from the B cell integration cluster was located on chromosome 21 and acted as a promoter of both inflammation and upregulation of salient oncogenic microRNAs (oncomiRs) in many human cancers [12]. miR-155 was an important biomarker for understanding the molecular mechanisms and etiology of various diseases [13, 14]. miR-155 was mainly expressed in the thymus and spleen [13]. miR-155 overexpression has been found to regulate several cancer-related pathways involved in cell growth, invasion, migration, stemness, and angiogenesis [15].

In recent years, many studies had shown that miR-155 played a regulatory role in lung cancer [16], kidney cancer [17], breast cancer [18, 19], colorectal cancer [20], and other tumors by targeting Forkhead box O3 (FOXO3). In addition, Yadav et al. [21] considered that miR-155 increased the tumorigenic properties of cancer cells through downregulation of Ubiquilin (UBQLN) in lung cells. Kong et al. [22] showed that miR-155 regulated the VHL-HIF-1*α*-VEGF pathway to induce tumor angiogenesis and metastasis. Overexpression of miR-155 induced tumor angiogenesis and promoted breast tumor growth through targeting and downregulating the Von Hippel-Lindau (VHL) tumor suppressor. It has been proposed by Lu et al. [23] that miR-155 participated in the pathogenesis of gastric adenocarcinoma and promoted the growth of gastric adenocarcinoma by regulating the IGF-1/IGF-1R signaling pathway. Kono et al. [24] demonstrated that upregulation of miR-155 affected the proliferation and invasion of gallbladder cancer (GBC) cells, which indicated a poorer prognosis for GBC patients, that is why miR-155 became a prognostic marker and therapeutic target for GBC. miR-155 was found to target the PTEN-PI3K-AKT signaling pathway to promote the proliferation of nasopharyngeal carcinoma cells (NPC cells) and inhibit the apoptosis of NPC cells, which turned into a new target for the treatment of NPC [25]. Upregulated S100 calcium-binding protein P (S100P) was associated with the pathogenesis of cancer such as human colon cancers since its discovery in 1992 [26]. Additionally, in a study by Onyeagucha et al. [26], miR-155 promoted the tumorigenic phenotype in colon cancer cells, which could be modulated by the S100P/RAGE signaling pathway. Specifically, the miR-155 level in colon cancer cells was not only upregulated by enforced S100P expression but depended on the receptor of advanced glycation end products (RAGE). Hence, the inhibition of S100P, RAGE, or miR-155 could suppress colon cancer growth and metastasis [26]. Tili et al. [27] showed that miR-155 allowed tumor development and progression via simultaneously targeting tumor suppressor genes and inducing a mutator phenotype.

In conclusion, reducing the endogenous levels of miR-155 with drugs or herbs should be a key role in the treatment of inflammation-related cancers.

## 3. miR-155 and Inflammation

Studies had proved that miRNAs delivered by exosomes could regulate the inflammatory response of endotoxins, while endogenous miRNAs were functionally transferred between immune cells and constituted a mechanism for regulating the inflammatory response [28]. miRNAs with different regulations were important for the epigenetic switch from nontransformed to cancer cells. During this process, the signal transducer and activator of transcription 3 (STAT3) was not only a downstream target of interleukin-6 (IL-6) but also a part of the positive feedback loop that underlays the epigenetic switch between inflammation and cancer [29]. It was further shown that the transient expression of either miRNAs could induce the epigenetic switch [29]. miR-155 has been confirmed to be a major regulator of inflammation and immune response.

miR-155 was a nuclear factor-*κ*B- (NF-*κ*B-) dependent miRNA. Yang et al. [30] speculated that the increased expression of miR-155 might restrain the NF-*κ*B signaling pathway to effectively reduce IL-1*β*-induced apoptosis, inflammation, and oxidative stress in rat nucleus pulposus cells. In addition, Xie et al. [31] speculated that inhibiting the expression of miR-155-5p might regulate the NF-*κ*B signaling pathway to upregulate FNDC3B, promote the proliferation of chondrocytes induced by IL-1*β*, and inhibit cell apoptosis. Liu et al. [32] selected the miR-155/NF-*κ*B signaling pathway to affect the changes of inflammatory factors in neonatal pig acute respiratory distress syndrome (ARDS) and, moreover, speculated that miR-155 might be a potential target for eliminating the inflammatory response after neonatal pig ARDS.

Furthermore, Zhang et al. [33] indicated that miR-155 knockdown improved nerve function and restrained inflammation by targeting MafB, as well as reducing cerebral ischemia-reperfusion injury (CIRI), becoming a new target for the treatment of CIRI. Korotkov et al. [34] considered that miR-155 promoted neuroinflammation via astrocyte activation, and miR-155 could be involved in secondary brain injury after traumatic brain injury (TBI). Additionally, it has been reported that miR-155 antagomir modulated Th17/Treg cell balance through Jarid2/Wnt/*β*-catenin to prevent DSS-induced colitis in mice [35]. Moreover, miR-155 was found to participate in the inflammatory response of ulcerative colitis (UC) by regulating the TLR9 signaling pathway [36]. Jin et al. [37] established that overexpression of miR-155, in the gouty synovial fluid mononuclear cells, decreased the levels of phosphatidylinositol 3,4,5-trisphosphate 5-phosphatase 1 (SHIP1) and promoted the production of monosodium urate (MSU) monohydrate-induced proinflammatory cytokines, such as tumor necrosis factor-*α* (TNF-*α*) and IL-1*β* [37]. It has been proved that miR-155 stimulated rheumatoid arthritis fibroblast-like synovial cell proliferation and inflammatory cytokine secretion by targeting FOXO3a [38]. While Jing et al. [39] found that long intergenic nonprotein encoding long-chain RNA p53-induced transcript (lncRNA LINC-PINT) increased the expression of suppressor of cytokine signaling-1 (SOCS-1) by sponging miR-155-5p and inhibited the activation of extracellular signal-regulated kinase (ERK) signaling pathway in rheumatoid arthritis synovial fibroblasts induced by TNF-*α* to treat rheumatoid arthritis (RA). What is more, research have shown that the proinflammatory effect of miR-155 contributed to liver fibrosis and alcohol-induced steatohepatitis, and miR-155 could also aggravate liver I/R damage and liver cell hypoxia/reoxygenation damage by depressing the expression of SOCS-1 [40, 41].

miR-155 established an important role in viral infections, particularly DNA viruses [42]. miR-155 could inhibit the host immune response to promote virus replication in the body [43]. In support of this hypothesis, Pareek et al. [44] reported that miR-155 was involved in the replication process of the encephalitis virus, as overexpression of miR-155 could promote the replication of Japanese encephalitis virus in human microglia clone 3 (CHME3). Zhai et al. [45] found that Brona virus infection controlled the expression of type I interferon by restraining miR-155 for protecting its own replication process and ensuring continuous infection. Bhela et al. [46] supported that miR-155 was an effective target to control keratitis caused by the sporangia virus. Furthermore, miR-155 limited the production of West Nile virus (WNV) in mouse and human cells and protected mice from lethal WNV infection, with the result that Natekar et al. [47] concluded miR-155 affected the pathogenesis and drug resistance of WNV and regulated antiviral cytokines as well.

Taken together, miR-155 was a critical role in inflammatory disease. miR-155 worked in a variety of cells or diseases by targeting different objective genes. We summarized the information of miR-155 in different inflammatory cells in Table 1.

### 3.1. miR-155 and T Cell

T cells were central to regulating the adaptive immune response for specific antigens, including gamma delta (*γδ*) and alpha-beta (*αβ*) T cells. Based on the cell surface expression of the coreceptor molecules CD4 and CD8, *αβ* T cells were further categorized. CD4^+^ T cells had low cytotoxic activity and promoted activating and modulating other immune cells to initiate the body's response to invading microorganisms, while CD8^+^ T cells, known as T cytotoxic (Tc) cells, could destroy or kill cells infected by foreign invading microorganisms [48, 49]. In chronic inflammatory diseases, T lymphocytes used their memory properties and high cytokine production capacity to control and regulate the host's response [50]. Specifically, T lymphocytes could be divided into a variety of T helper (Th) subgroups, including Th1, Th2, Th17, and regulatory T cells (Treg), exhibiting anti-inflammatory or proinflammatory properties [51]. Th17 cells could induce autoimmunity, promote tissue inflammation, launch innate immunity, and mediate the occurrence and development of inflammation, tumors, autoimmune diseases, etc. [52, 53]

On one hand, many reports have shown that miR-155 was closely associated with Th17. In a study by Escobar et al. [54], miR-155 regulated by STAT3 was therapeutic targets for Th17-mediated inflammatory disorders. In a further study, it has been identified that miR-155 regulated the differentiation process of Th17 cells and Th9 cells through the c-Maf pathway to promote wound healing [55]. Additionally, inhibition of miR-155 could improve the intestinal barrier function of *β*-lactoglobulin (*β*-Lg) allergic mice by reducing the levels of IL-6 and IL-21 and increasing TGF-*β*1 and affect the differentiation and function of Th17 cells involved in the Jarid2/notch1 pathway [56]. miR-155-5p could significantly aggravate the rhinitis symptoms of allergic rhinitis (AR) mice, with the result that Tang et al. [57] speculated it might be related to affecting the expression of Foxp3 and ROR*γ*t and regulating the immune balance of Treg/Th17. Furthermore, Kun et al. [58] proved that Sanziyangqin Decoction regulated Th17/Treg balance by suppressing miR-155-5p to treat bronchial asthma.

On the other hand, miR-155 was found to be connected with Th2. Okoye et al. [59] identified that miR-155-regulated sphingosine-1-phosphate receptor 1 (S1pr1) in the pathogenesis was very important to Th2-mediated allergy. Th2-mediated airway inflammation required S1pr1, and the downregulation of S1pr1 was needed for lymphocyte egress from lymphoid tissue. miR-155 targeted S1pr1 and directly regulated S1pr1 to control Th2 cell migration [59]. In addition, studies have shown that the expression of miR-155 was increased in patients with allergic dermatitis, while in miR-155-deficient mouse models, both Th2 cells and inflammatory eosinophils were reduced. It can be inferred from the above phenomenon that miR-155 was a therapeutic target for allergic dermatitis [60, 61].

On the third, miR-155 was of great importance in regulating interferon (IFN) responsiveness and CD8^+^ T cell responses against pathogens in vivo. Gracias et al. [62] showed that miR-155 was upregulated in the primary effector and effector memory CD8^+^ T cells but downregulated in naive and central memory cells. While Bhela et al. [63] proposed that miR-155 modulated the failure and long-term persistence of CD8^+^ T cells during chronic infection by inhibiting Fos-like antigen 2 (Fosl2) in the activator protein-1 (AP-1) pathway. Furthermore, Huffaker et al. [64] demonstrated that miR-155 promoted immune responses, especially IFN*γ* responses, through a mechanism involving the repression of SHIP1, and miR-155 played a critical role in the reciprocal regulation of CD4^+^ and CD8^+^ T cell-mediated antitumor immunity in the regulation of antitumor immune responses [64]. Varikuti et al. [65] investigated in the resolution of visceral leishmaniasis (VL) and found that miR-155 promoted CD4^+^ Th1 response and IFN-*γ* production by targeting the suppressor of SOCS-1 and SHIP1. In addition, a further study proved that in activated CD4^+^ T cells, miR-155 targeted SHIP1 and IFN-gRa to control the severity of stromal keratitis (SK) [66] (Table 1).

### 3.2. miR-155 and B Cell

B lymphocytes were a critical component of the adaptive immune system as they were the source of humoral immunity and contributed to pathological immune responses via the secretion of cytokines, costimulation of T cells, antigen presentation, and the production of autoantibodies, which suggested that B cells were important for the effective regulation of the immune system and used a wide array of immunosuppressive mechanisms [67, 68].

As an antibody-mediated signal regulator, miR-155 participated in the process from the regulation of B cell function to muscle regeneration and tissue renewal, as well as played a key regulatory role in hematopoiesis and B cell differentiation [69]. Marsolier et al. [69] demonstrated that miR-155 was the crossroads between infection, regulatory circuits, and transformation. During the process that parasite infection regulated the circuitry of host leukocytes, miR-155 targeted DET1, an evolutionarily conserved factor involved in c-Jun ubiquitination, to stable c-Jun and active B cell integration cluster (BIC) transcript [69].

Additionally, Higgs et al. [70] reviewed that miR-155 was a noncoding transcript expressed in activated B cells, T cells, monocytes, and macrophages. And miR-155 was produced from the processing of the BIC, which was induced by avian leukosis virus, simultaneously activated by promoter insertion at a retroviral integration site on chromosome 21q21 in B cell lymphomas, so that miR-155 absence led to defects in hematopoietic development [70]. A further study found that downregulation of miR-155 could promote B-lymphoma cell apoptosis and delay the formation of xenograft tumors in nude mice [71]. Moreover, Sandhu et al. [72] investigated that miR-155 could induce high-grade lymphoma/leukemia and pre-B cell proliferation and ensured that miR-155 caused disruption of the B cell lymphoma 6 (BCL6) transcriptional machinery and upregulated the survival and proliferation genes. As a key transcriptional repressor and protooncogene, BCL6 could promote cell survival and proliferation like the inhibitor of differentiation 2 (Id2), IL-6, and c-Myc, which was indirectly regulated by miR-155 through the upregulation of Mxd1/Mad1 [73]. While miR-155 directly targeted histone deacetylase 4 (HDAC4), a corepressor partner of BCL6, and ectopic expression of HDAC4 reduced miR-155-induced proliferation and clonogenic potential, simultaneously increased apoptosis in human-activated B cell-type that diffuse large B cell lymphoma (DLBCL) cells [73].

In immune cells, proliferative immune response disorder was an important factor in inducing DLBCL [74]. During these years, many studies have reported that miR-155 was directly bound up with the occurrence and development of DLBCL. Therefore, miR-155 was expected to become a treatment target for DLBCL patients to improve the treatment effect on patients and the prognosis [75, 76]. To support this hypothesis, a further study demonstrated that miR-155 promoted B cell lymphoma cell proliferation and inhibited cell apoptosis through targeting inhibition of FOXO3, and both miR-155 overexpression and FOXO3 low-expression were related to poor prognosis in patients with DLBCL [77, 78]. Additionally, Huang et al. [78] provided that overexpression of miR-155 downregulated both the transcription and translation of p85*α*, which was a negative regulator of the phosphatidylinositol 3-kinase- (PI3K-) AKT pathway in DLBCL, while Li et al. [79] proved that miR-155 could also directly downregulate the specific human germinal center-associated lymphoma (HGAL) gene in the germinal center, thereby reducing the activation of ras homolog family member A (RhoA), increasing the migration ability of lymphoma cells, and promoting the metastasis and infiltration of DLBCL. Moreover, Pedersen et al. [80] demonstrated that miR-155 targeted SHIP1 to promote TNF-*α*-dependent B cell lymphomas growth, and upregulated miR-155 and consequently downregulated SHIP1 expression brought about autocrine stimulation by the proinflammatory cytokine TNF-*α* in DLBCL. They further researched that miR-155 was a TNF-*α*-inducible transcript, and the expression levels of SHIP1 and miR-155 were valuable prognostic indicators in DLBCL, so that anti-TNF-*α* therapy could be used as a novel and immediately accessible (co)treatment for DLBCL [80].

The interaction of the Epstein-Barr virus (EBV) and B lymphocytes in infection, immunity, and disease was well known [81]. Linnstaedt et al. [82] suggested that miR-155 played a key role in B cell immortalization by EBV. More specifically, when primary human B cells were infected by EBV, they were turned into indefinitely proliferating lymphoblastoid cell lines (LCLs), which acted as a model for lymphomagenesis, phenotypically similar to EBV-positive DLBCLs, while miR-155 was the most highly expressed miRNA in LCLs and selectively restrained the growth of both LCLs and the DLBCL cell line IBL-1 [82]. In addition, a study by Hatton et al. [83] found that EBV latent membrane protein 1 (LMP1) regulated miR-155 and its targeted-FOXO3a in B cells by activating PI3K p110*α*, which could be a reasonable therapeutic target and biomarker for EBV^+^ B cell lymphoma. Another study proposed that overexpression of miR-155 and SOCS-1 was the characteristic of primary Sjögren's syndrome (pSS) [84]. It was further shown that EBV infection seemed to contribute to the local growth and differentiation of Sjögren-specific autoreactive B cells [85]. By using antagomirs to inhibit the activity of miR-155 and miR-125b in B cells, Farroni et al. [86] got the result that inhibiting miR-155 reduced the number of plasma cells (PCs) in healthy donors (HD) and down syndrome (DS), confirming that abnormal miR-155 and miR-125b were associated with impaired B cell response in DS (Table 1).

### 3.3. miR-155 and DC

Dendritic cells (DCs) were antigen-presenting cells (APCs) for immune control, normally derived from bone marrow precursors distinct from monocytes, and controlled the results of innate immune response and adaptive immune response through phenotypic and functional transformation. DCs were key regulators of T cell-mediated immune responses, which helped to recognize pathogens and tumors, and played an important role in cancer immunity, transplantation immunity, autoimmune response, and infection immunity [87–89].

A study reported that the upregulation of miR-155 in DC could lead to the destruction of T cell tolerance through the negative regulation of SHIP1 [90]. Additionally, Jia et al. [90] demonstrated through experiments in endometrial cancer mice that miR-155 inhibited the translation of p38, impaired the function of dendritic cells, and reduced their ability to interfere with tumor growth. Furthermore, Gao et al. [91] observed that suppression of miR-155 in DCs could be used as a viable therapeutic strategy for the prevention and treatment of allograft rejection in the clinical setting of transplantation. In detail, miR-155 was induced during DC differentiation, and its expression depended on the TLR4/Myd88/NF-*κ*B signal; inhibition of miR-155 expression in DCs downregulated lipopolysaccharide- (LPS-) induced DC maturation, along with reduced ability to stimulate allogeneic T cell proliferation, so that miR-155 could promote DC maturation and regulated its ability for antigen presentation and induction of alloreactive T cell activation [91]. Taken together, blocking miR-155 had a great function in improving the treatment of DCs.

Tolerant dendritic cells (tDCs) could tolerate T cell immunity and induce Treg to establish immune tolerance, which could be used as a therapeutic target for posttransplantation or autoimmune diseases [92, 93]. Wu et al. [94] found that overexpression of metastasis-associated lung adenocarcinoma transcript (MALAT1) could induce tDCs and immune tolerance via the miRNA-155/DC-SIGH/IL-10 axis.

Dendritic cell-specific intercellular adhesion molecule-3 grabbing nonintegrin (DC-SIGN) was an innate immune receptor, mainly expressed in dendritic cells and macrophages, involved in the pathogen of dendritic cells recognition and antigen presentation, participating in the maintenance of immunosuppression during transplantation, tumor growth, and pathogenic infection [95, 96]. According to reports, PU and miR-155 were involved in the maturation of human DC. By directly regulating Pu.1, miR-155 could indirectly target DC-SIGN, thereby regulating DC function and allogeneic immunity [97, 98]. In mouse and human microchip experiments [99], the increase of miR-155 expression was positively correlated with the maturation of monocytes and mouse bone marrow-derived dendritic cells (BMDC), and miR-155 also anticipated the expression of MHCII during the process of granulocyte-macrophage colony-stimulating factor- (GM-CSF-) induced dendritic cell development and lipopolysaccharide-induced dendritic cell maturation, so Young et al. [99] concluded that the immune function of dendritic cells was reduced when miR-155 was absent. A further study found that bone mesenchymal stem cells (BMSCs) with high expression of miR-155 could induce the formation of tolerant DCs by inhibiting NF-*κ*B and AKT pathways, which strengthened the immune regulation ability of BMSCs [100]. miR-155 was emerging as a new target in AS. Indeed, Yan et al. [101] showed that oxidized low-density lipoprotein (oxLDL), a pathogenic role in the occurrence and development of atherosclerosis (AS), could promote miR-155 expression in DCs through the SRA receptor and JAK2 signaling pathway. Interestingly, oxLDL could also be suppressed by miR-155 through the negative feedback loop miR-155-JNK-SRA-miR-155.

For the reason that the enhancement of miR-155 induced the increase of CD40 expression in symptomatic pDCs, Yan et al. [102] believed that regulation of miR-155 could help alleviate the excessive activation of TLR7 in systemic lupus erythematosus (SLE) pDCs. In addition, Chen et al. [103] found miR-155 promoted the migration of DC to the ATP release site and also activated the inflammasome. Inhibiting the proinflammatory miR-155 helped to reduce acute graft-versus-host disease (GVHD), which limited the success of allogeneic hematopoietic cell transplantation (alloc-hct) in the treatment of acute leukemia [103]. Monocyte-derived dendritic cells (MDDCs) were the first line of defense and are known as the initial target of infection in human immunodeficiency virus (HIV) and other infections. Napuri et al. [104] showed that, as the maturation of DCs led to less susceptibility to HIV-1 infection, suppressing miR-155 could prevent the maturation of DCs, resulting in increased DC-SIGN expression and susceptibility to HIV-1 infection. Therefore, miRNA-based therapeutic strategies could be a novel way to treat HIV-1 infection [104]. Additionally, it has been demonstrated that the increased expression of miR-155, targeting NF-*κ*B p65 and BCL-10, could inhibit the inflammatory response of human MDDCs induced by *Candida albicans* [105]. Etna et al. [106] found *Mycobacterium tuberculosis* (Mtb) induced host miR-155 to target recombinant human autophagy-related 3 (ATG3) and impaired autophagosome formation in infected DCs to manipulate autophagy, proposing that normal autophagy could be reconstructed by antisense miR-155 molecules to treat tuberculosis. Meanwhile, Li et al. [107] found that N-3-oxododecanoyl-L-homoserine lactone (3-oxo-C12-HSL) could be regulated by controlling the expression of miR-155, which was an important signal molecule secreted by *Pseudomonas aeruginosa* (PA) (Table 1).

### 3.4. miR-155 and Macrophage

Macrophages, long-lived innate immune cells of the mononuclear phagocyte system and present throughout the human body from distinct organs to tissues, totally could be considered prototypic immune cells. Macrophages participated in tissue homeostasis and induction of inflammatory reactions towards pathogens [108]. Macrophages were mainly divided into two types: proinflammatory type-1 macrophages (M1-type macrophages) and anti-inflammatory type-2 macrophages (M2-type macrophages). M1-type macrophages mainly produced proinflammatory factors and reactive oxygen species (ROS), but excessive M1 macrophages could cause inflammation and tissue damage. On the contrary, M2-type macrophages mainly produced anti-inflammatory factors, inhibited inflammation, promoted tissue repair, increased the response to fungal infections by reducing autophagy, and promoted other immune cells in an adaptive mode [109–111].

As upregulation of miR-155 caused excessive inflammation to bring about heart defects by promoting M1 polarization, it was further shown that gold nanoparticles (AuNPs) in macrophages specifically inhibited the expression of miR-155, promoting M2 polarization, inhibiting inflammation, and restoring cardiac function [112]. Additionally, Teng et al. [113] reported that anti-miR-155 polarized M2 by downregulating BCL6 in the cell, and baicalein quickly kept in inflammation mainly by restraining the secretion of NF-*κ*B after administration. As a result, the targeted codelivery of anti-miR-155 and anti-inflammatory baicalein was used to inhibit inflammation and treat AS. A further study showed that overexpression of miR-155 in macrophages mediated by lentivirus against SOCS-1 enhanced their inflammatory response by LPS [114]. Moreover, targeting miR-155 was accepted to halt AS, because miR-155 deficiency reduced inflammatory responses of macrophages and attenuated AS; consequently, miR-155 enhanced macrophage cholesterol efflux and contributed to an antiatherogenic leukocyte profile [115]. Several researches had shown that miR-155HG regulated the expression of TNF-*α*, IL-1*β*, IL-10, and IL-12, together with GM-CSF-mediated polarization of M1/M2 macrophages in the progression of chronic obstructive pulmonary disease (COPD) [116]. Especially, overexpression of miR-155HG promoted GM-CSF-induced polarization of M1 macrophages and the release of proinflammatory cytokines, while downregulation of miR-155HG could inhibit the polarization of M1 macrophages and increase the polarization of M2 macrophages [117]. Furthermore, macrophages could damage the arterial wall in that the production of collagen was broken down by matrix metalloproteinases. In support of this opinion, Zhang et al. [117] demonstrated that inhibition of miR-155 could reduce the inflammation and matrix proteolysis in the abdominal aortic aneurysm (AAA) by regulating the infiltration of macrophages; consequently, miR-155 became a new intervention target for AAA.

Tumor-associated macrophages (TAMs), a prominent inflammatory cell population, are mainly polarized into “selectively activated” M2-like macrophages. miR-155 targeted CCAAT/enhancer-binding protein (C/EBPb) to transform the original tumor M2 TAMs into antitumor M1 macrophages. Additionally, it has been demonstrated that sPEG/GLC could successfully import miR-155 into TAMs and effectively repolarize the original tumor M2 TAMs into antitumor M1 macrophages, so that sPEG/GLC/miR-155 could induce antitumor immune responses [118]. In a study by Bala et al. [119], they pointed out that chronic alcohol consumption increased miR-155 in macrophages via NF-*κ*B, and upregulated miR-155 contributed to alcohol-induced elevation in TNF-*α* production through increased mRNA stability in alcoholic liver disease (Table 1).

## 4. Summary and Perspective

**Figure 2:**
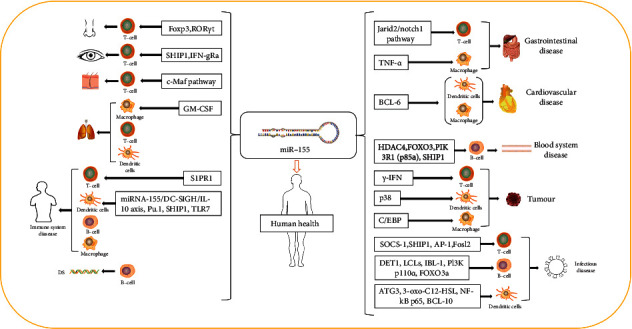
Main roles of miR-155.

In conclusion, miRNAs can not only constitute a mechanism for regulating the inflammatory response but play a function similar to oncogene or tumor suppressor gene in the tumor process [120]. As one of the best-characterized miRNAs, miR-155 is processed from the non-protein-coding transcript of the BIC gene, closely related to B cells, T cells, DCs, and macrophages. Especially, miR-155 has great effects on the normal function of B cells, T cells, and DCs; simultaneously, its expression is increased during B cell, T cell, macrophage, and DC activation [121]. Moreover, miR-155 is also a multifaceted regulator of proliferation, chemoresistance, and apoptosis. The functions mentioned above make miR-155 an attractive therapeutic target in physiological and pathological processes such as cancer, inflammation, infection, and immunity. The miR-155 acts in different inflammatory cells are presented in Table 1. The main roles of miR-155 are introduced vividly in Figure 2. Through a literature survey, we have found that suppression of miR-155 is widely applied in human diseases, which prompts a more novel and effective therapy. However, it is a pity that there is still little connection between miR-155 and traditional Chinese medicine. But in terms of the recent experiments, applying traditional Chinese medicine to target miR-155 to control inflammatory cells has a good curative effect in regulating the body's immune mechanism. Thus, we can conclude that targeting miR-155 can be used as a therapeutic strategy for the diagnosis, prevention, and treatment of various diseases in both Chinese and Western medicine in the future.

## Figures and Tables

**Table 1:** Biological function of miR-155 in different inflammatory cells.

Disease or biological process	Inflammatory cell	Function of miR-155	Reference
Th17-mediated inflammatory disorders	Th17 cell	Therapeutic targets	54
Wound healing	Th17 cell	Regulate Th17 cells and Th9 cells through the c-Maf pathway	55
	Th9 cell		
*β*-Lg allergy intestinal barrier function	Th17 cell	Reduce IL-6 and IL-21, increase TGF-*β*1, and regulate the Jarid2/notch1 pathway	56
Allergic rhinitis (AR)	Th17 cell	Affect Foxp3 and ROR*γ*t	57
	Treg		
Bronchial asthma	Th17 cell	Therapeutic targets	58
	Treg		
Th2-mediated allergy	Th2 cell	Target S1PR1 and regulate Th2 cell migration	59
Allergic dermatitis	Th2 cell	Therapeutic targets	60,61
Inflammation	CD8^+^ T cell	Regulate IFN responsiveness and CD8^+^ T cell responses	62
Chronic infection	CD8^+^ T cell	Inhibit Fosl2 in the AP-1 pathway	63
Antitumor immune responses	CD4^+^ T cell	Promote IFN*γ* responses	64
	CD8^+^ T cell		
Visceral leishmaniasis (VL)	CD4^+^ T cell	Target SOCS-1 and SHIP1	65
Stromal keratitis (SK)	CD4^+^ T cell	Target SHIP1 and IFN-gRa	66
Parasite infection	B cell integration cluster (BIC)	Target DET1 and promote activity of the B cell integration cluster (BIC) transcript	69
Hematopoietic development	B cell	Promote hematopoietic development	70
Diffuse large B cell lymphoma (DLBCL)	B cell	Therapeutic targets	71
Leukemias	Pre-B cell	Directly target HDAC4	72
Diffuse large B cell lymphoma (DLBCL)	Diffuse large B cell lymphoma (DLBCL) cells	Target inhibition of FOXO3	76,77
		Downregulate both the transcription and translation of PIK3R1 (p85*α*)	78
		Downregulate the specific HGAL gene	79
TNF-*α*-dependent B cell lymphoma growth	Diffuse large B cell lymphoma (DLBCL) cells	Target SHIP1 to promote TNF-*α*-dependent B cell lymphoma growth	80
Epstein-Barr virus (EBV)	B cell	Inhibit the growth of both LCLs and the DLBCL cell line IBL-1	82
EBV^+^ B cell lymphoma	B cell	Activate PI3K p110*α* and target FOXO3a	83
Primary Sjögren's syndrome (pSS)	B cell	Therapeutic targets	84
Down syndrome (DS)	B cell	Therapeutic targets	86
T cell tolerance destruction	Dendritic cell (DC)	Target SHIP1	89
Endometrial cancer	Dendritic cell (DC)	Inhibit the translation of p38	90
Allograft rejection in the clinical setting of transplantation	Dendritic cell (DC)	Therapeutic strategy for prevention and treatment	91
Immune tolerance	Tolerant dendritic cells (tDCs)	miRNA-155/DC-SIGH/IL-10 axis	94
Allogeneic immunity	Dendritic cell-specific intercellular adhesion molecule-3 grabbing nonintegrin (DC-SIGN)	Regulate Pu.1	97,98
Immune function	Bone marrow-derived dendritic cells (BMDC)	Therapeutic targets	99
Atherosclerosis (AS)	Dendritic cell (DC)	Inhibit oxLDL through the negative feedback loop miR-155-JNK-SRA-miR-155	101
Systemic lupus erythematosus (SLE)	Dendritic cell (DC)	Alleviate the excessive activation of TLR7	102
Graft-versus-host disease (GVHD)	Dendritic cell (DC)	Promote the migration of DC to the ATP release site and activate the inflammasome	103
HIV-1 infection	Monocyte-derived dendritic cells (MDDCs)	Prevent the maturation of DCs and increase susceptibility to HIV-1 infection to reduce HIV-1 replication	104
The inflammatory response of human MDDCs induced by Candida albicans	Monocyte-derived dendritic cells (MDDCs)	Target NF-*κ*B p65 and BCL-10	105
Tuberculosis	Dendritic cell (DC)	Target ATG3	106
*Pseudomonas aeruginosa* (PA)	Dendritic cell (DC)	Control N-3-oxododecanoyl-L-homoserine lactone (3-oxo-C12-HSL)	107
Heart disease	Macrophages	Promote M2 polarization, inhibit inflammation, and restore cardiac function	112
Atherosclerosis (AS)	Macrophages	Downregulate BCL-6 to polarize M2	113
Inflammatory response	Macrophages	Reduce inflammatory responses of macrophages and attenuate atherogenesis, enhance macrophage cholesterol efflux, and lead to an antiatherogenic leukocyte profile	114
Chronic obstructive pulmonary disease (COPD)	Macrophages	Regulate GM-CSF-mediated polarization of M1/M2 macrophages	115
Abdominal aortic aneurysm (AAA)	Macrophages	Regulate the infiltration of macrophages to reduce the inflammation and matrix proteolysis	117
Antitumor immune responses	Macrophages	Target C/EBPb to transform the original tumor M2 TAMs into antitumor M1 macrophages	118
Alcoholic liver disease	Macrophages	Contribute to alcohol-induced elevation in TNF-*α* production	119

## Data Availability

The data supporting this review are from previously reported studies and datasets, which have been cited. The processed data generated or analyzed during this study are included in this article.
